# Highly Pathogenic H5N1 Influenza A Virus Spreads Efficiently in Human Primary Monocyte-Derived Macrophages and Dendritic Cells

**DOI:** 10.3389/fimmu.2018.01664

**Published:** 2018-07-17

**Authors:** Veera Westenius, Sanna M. Mäkelä, Ilkka Julkunen, Pamela Österlund

**Affiliations:** ^1^Expert Microbiology Unit, Department of Health Security, National Institute for Health and Welfare, Helsinki, Finland; ^2^Institute of Biomedicine, University of Turku, Turku, Finland

**Keywords:** influenza A virus, avian influenza, macrophages, dendritic cells, viral replication, innate immunity

## Abstract

Influenza A viruses cause recurrent epidemics and occasional global pandemics. Wild birds are the natural reservoir of influenza A virus from where the virus can be transmitted to poultry or to mammals including humans. Mortality among humans in the highly pathogenic avian influenza H5N1 virus infection is even 60%. Despite intense research, there are still open questions in the pathogenicity of the H5N1 virus in humans. To characterize the H5N1 virus infection in human monocyte-derived macrophages (Mɸs) and dendritic cells (DCs), we used human isolates of highly pathogenic H5N1/2004 and H5N1/1997 and low pathogenic H7N9/2013 avian influenza viruses in comparison with a seasonal H3N2/1989 virus. We noticed that the H5N1 viruses have an overwhelming ability to replicate and spread in primary human immune cell cultures, and even the addition of trypsin did not equalize the infectivity of H7N9 or H3N2 viruses to the level seen with H5N1 virus. H5N1 virus stocks contained more often propagation-competent viruses than the H7N9 or H3N2 viruses. The data also showed that human DCs and Mɸs maintain 1,000- and 10,000-fold increase in the production of infectious H5N1 virus, respectively. Both analyzed highly pathogenic H5N1 viruses showed multi-cycle infection in primary human DCs and Mɸs, whereas the H3N2 and H7N9 viruses were incapable of spreading in immune cells. Interestingly, H5N1 virus was able to spread extremely efficiently despite the strong induction of antiviral interferon gene expression, which may in part explain the high pathogenicity of H5N1 virus infection in humans.

## Introduction

Influenza A viruses are one of the most important viral pathogens annually infecting even 5–10% of the human population and causing an estimated 250,000–500,000 deaths worldwide. Occasionally, influenza A viruses cause global pandemics of which the most devastating one was the pandemic caused by Spanish flu in 1918 leading up to 50 million deaths (1). The segmented genome of influenza A viruses confers evolutionary advantages and influenza A viruses have a great ability to evolve by two different mechanisms, antigenic drift and shift. All pandemics in the twentieth century have been of avian origin (2, 3). Avian influenza A viruses circulate continuously in birds and occasionally they have caused infections in humans. The highly pathogenic avian influenza (HPAI) H5N1 viruses have caused almost 900 human infections from 2003 until today and the mortality among humans has been over 50% (4). The H7N9 virus, which emerged in humans in 2013, has a surprising character as it causes only a mild infection in domestic poultry but an HPAI-like disease in humans. In the beginning of 2017, a human infection was detected with H7N9 virus possessing a multi-basic HA1-HA2 cleavage site motif in the hemagglutinin (HA) molecule characteristic of HPAI viruses (5). Thus far, there have been over 1,500 human infections caused by the H7N9 virus with a mortality rate reaching 40% (4, 6). Therefore, these avian influenza virus strains pose a serious risk for a pandemic. Despite active research, it is not completely clear which factors in H5N1 or H7N9 viruses or in host responses contribute to a severe disease in humans.

After infecting the epithelial cells of respiratory tract, influenza A viruses can spread to alveolar dendritic cells (DCs) and macrophages (Mɸs) which reside in the immediate proximity of the epithelium (7). DCs and Mɸs are the key cell types to orchestrate host immune responses (8, 9). Typically influenza A virus induces antiviral responses in immune cells by inducing type I and III interferons (IFNs) which are known to inhibit virus replication and propagation (10–12). Both alveolar and monocyte-derived Mɸs as well as DCs, including monocyte-derived DCs (moDCs), express receptors for both avian-adapted (α-2,3-linked sialic acids) and human-adapted (α-2,6-linked sialic acids) influenza A viruses (13–16). The viral HA binds to sialylated host cell surface receptor molecules and mediates virus entry. Influenza viruses enter the cells mainly *via* endocytosis and the fusion of viral and endosomal membranes. For the fusion to happen the precursor form of the HA, HA0 has to be cleaved into HA1 and HA2 subunits by host cells proteases. The membrane fusion mediated by the mature form of the HA occurs at low pH which enables the release of the segmented viral genome into the cytoplasm. The genome of the influenza virus is structured in eight separate viral ribonucleoprotein (vRNP) complexes which are transported into the nucleus for the transcription and replication of the virus. The viral proteins are translated in the cytoplasm but the viral proteins are assembled into vRNPs in the nucleus. Newly synthesized vRNPs are exported to the cytoplasm, virus particles are assembled at the cell membrane, and progeny virus particles bud out of the cell. All eight vRNAs have to be packed into a virion to produce infective progeny viruses and the infection to be productive. The mechanism behind the genome packaging is not fully understood but it is believed that influenza A virus packs its vRNAs in a specific manner by a selective packaging mechanism (17). Some studies suggest that most influenza A virus particles are noninfectious since they express incomplete set of viral gene segments and are incapable of inducing a secondary infection (18). However, three-dimensional analysis of the virions has shown that at least 80% of virions have all eight RNPs packaged (19). In addition, it is known that there are differences between various seasonal influenza virus strains in their ability to cause a productive infection (18) but the comparison between avian influenza and seasonal influenza virus strains in primary human cells have remained poorly characterized.

Previously, we have shown that human moDCs are susceptible to the avian influenza virus infection (12, 20). In this study, we show that the highly pathogenic H5N1 influenza A viruses can efficiently replicate and produce new infective particles in human primary moDCs and Mɸs and, despite the strong IFN-mediated antiviral responses induced by the infection, be able to spread throughout the whole immune cell culture. These results suggest that the excessive cytokine production (“cytokine storm”) induced by H5N1 infection may in fact be due to extremely efficient spread of the virus infection in the infection site leading to greatly enhanced cytokine gene expression.

## Materials and Methods

### Ethics Statement

The permission to import the human isolates of avian virus strains for research purposes was obtained from the Finnish Food Safety Authority (permission no 8634/0527/2012). Infective H5N1 and H7N9 viruses were handled strictly under Biosafety Level (BSL) 3 laboratory conditions at the National Institute for Health and Welfare (THL), Finland. Different virus subtypes were always handled in separate biosafety cabinets to avoid any possible creation of recombinant viruses. Adult human blood was obtained from anonymous healthy blood donors through the Finnish Red Cross Blood Transfusion Service (permission no 37/2016, renewed annually). Animal immunizations related to this study were approved by the Ethical Committee of the National Institute for Health and Welfare (permission no. KTL 2008-02).

### Cell Cultures

The buffy coats were obtained from healthy blood donors (Finnish Red Cross Blood Transfusion Service, Helsinki, Finland). Monocytes were purified from buffy coats as described previously (21). Human peripheral blood mononuclear cells were isolated by density gradient centrifugation over a Ficoll-Paque gradient (Amersham Biosciences).

To obtain monocytes for Mɸ differentiation, mononuclear cells were allowed to adhere onto plates or glass coverslips for 1 h at +37°C in RPMI 1640 (Sigma-Aldrich) supplemented with 0.6 µg/ml penicillin, 60 µg/ml streptomycin, 2 mM l-glutamine, and 20 mM HEPES. Nonadherent cells were removed by washing with phosphate-buffered saline (PBS), and the remaining monocytes were cultured in Mɸ serum-free medium (Life Technologies) supplemented with streptomycin and human recombinant granulocyte-macrophage colony-stimulating factor (rGM-CSF) (10 ng/ml; Nordic Biosite). The cells were differentiated into Mɸs for 7 days, with a change to fresh culture medium every 2 days.

To obtain moDCs, Percoll gradient (Amersham Biosciences) centrifugation was done after Ficoll-Paque gradient centrifugation. The fraction containing monocytes were collected and remaining T and B cells were depleted by using anti-CD3 and anti-CD19 magnetic beads (Dynal). Monocytes were allowed to adhere to plates (Sarstedt) for 1 h at +37°C in RPMI 1640 supplemented as described above. Non-adherent cells were removed by washing with PBS, and immature moDCs were generated by cultivating adherent monocytes in RPMI 1640 supplemented as described above and with 10% fetal calf serum (Integro), 10 ng/ml human rGM-CSF, and 20 ng/ml human recombinant interleukin-4 (R&D Systems). The cells were cultivated for 6 days, and fresh medium was added every 2 days.

In each experiment, cells from three to four different donors were cultured and used separately for infection experiments.

### Viruses

Human influenza A/Beijing/353/89 (H3N2) virus (originates from WHO Collaborating Centre for Reference and Research on Influenza, The Francis Crick Institute, UK) and human isolates of the avian influenza viruses A/Vietnam/1194/2004 (H5N1) and A/Hong Kong/156/1997 (H5N1) (Molecular Virology, Erasmus MC—Department of Viroscience, Rotterdam, Netherlands) and A/Anhui/1/2013 (H7N9) (WHO Collaborating Centre for Reference and Research on Influenza, The Francis Crick Institute, UK) were grown in the allantoic cavity of 10- to 11-day-old embryonated chicken eggs at +36°C for 2–3 days. Since the infectivity of influenza A viruses vary from one type of cell to another, the virus titers were determined by different assays. A hemagglutination titration was done with standard protocol using 0.5% turkey or guinea pig red blood cells. The infective virus titer in Madin–Darby Canine Kidney (MDCK) cells were determined by plaque assay. The viruses were serially diluted and inoculated to confluent MDCK cells on the 6-well plates for 1 h at 37°C. After incubation, cells were washed with PBS and covered with Avicel microcrystalline cellulose [Eagle-MEM containing 1.2% Avicel (#RC-591 NF, FMC BioPolymers)], 0.3% BSA, 2 μg/ml *N*-tosyl-l-phenylalanine chloromethyl ketone (TPCK) treated trypsin, 60 µg/ml streptomycin, 2 mM l-glutamine, and 20 mM HEPES. After incubation at 37°C for 1 day for supernatant samples and for 2 days for virus stocks Avicel was removed, cells washed with PBS, fixed with 4% paraformaldehyde (PFA), and stained with diluted crystal violet. The plaques were counted to obtain the concentration of infective viruses as plaque forming units (PFU)/ml.

The infectivity of the viruses was determined in Mɸs by immunofluorescence microscopy and in moDCs by flow cytometry. The number of infected cells with different virus dilutions was counted to obtain the concentration of infective virus particles as focus forming units per milliliter (FFU/ml). The multiplicity of infection (MOI) in Mɸs or moDCs is given according to the titers determined in Mɸs or moDCs, respectively (Table 1).

**Table 1:** Quantitation of H3N2, H5N1/1997, H5N1/2004, and H7N9 virus stocks.

	HA (turkey)	HA (guinea pig)	PFU/ml [Madin–Darby Canine Kidney (MDCK)]	FFU/ml [macrophage (Mɸ)]	FFU/ml [monocyte-derived DC (moDC)]
A/Beijing/353/89 (H3N2)	128	256	2 × 10^7^	3 × 10^7^	5 × 10^6^
A/Hong Kong/156/97 (H5N1)	256	256	2 × 10^8^	2.8 × 10^8^	1.2 × 10^8^
A/Vietnam/1194/04 (H5N1)	128	256	1 × 10^7^	7.8 × 10^8^	7.8 × 10^7^
A/Anhui/1/13 (H7N9)	1,024	1,024	2 × 10^8^	4.7 × 10^8^	1 × 10^7^

*The infectivity titers of viral stocks propagated in chicken eggs were determined in turkey and guinea pig red blood cells by hemagglutination assay (HA), in MDCK cells by the plaque forming assay (PFU), in human macrophages (Mɸs) and dendritic cells (moDC) by focus forming assay (FFU) with microscopy and flow cytometry, respectively*.

### Virus Infection Experiments

Macrophages and moDCs were infected with different MOI values of virus for different times, as indicated in the figures. Mɸs were differentiated on glass coverslips or cell culture plates, growth medium was removed, and virus dilution was added on cells. After 1 h at 37°C incubation, cells were washed with PBS, and fresh medium was added. For infection experiments in moDCs, the virus dilutions were added into cell cultures medium without changing the growth medium. For 6 h infectivity experiments in Mɸs and moDCs, cells were incubated with neuraminidase inhibitor, 20 nM oseltamivir carboxylate which is an active metabolite of oseltamivir phosphate (#RO0640802-002, Roche). All infection experiments with H5N1 and H7N9 viruses were performed in BSL-3 facility and only samples inactivated with validated methods were brought out from the BSL-3 laboratory.

### Immunofluorescence Microscopy

Macrophages were differentiated on glass coverslips, infected with different influenza A viruses at MOI values as indicated in the figures, incubated for 1 h at 37°C, washed with PBS, fresh Mɸ medium was added and incubated at 37°C with 5% CO_2_. At different times after infection, cells were washed, fixed with 4% PFA for 30 min at room temperature, washed, permeabilized with 0.1% Triton X-100 for 5 min, washed, and blocked with 0.5% BSA for 30 min. A/Beijing/353/89 (H3N2) virus-infected cells were stained with guinea pig antibody against influenza A virus H3N2 nucleoprotein (NP) (22). To visualize A/Vietnam/1194/2004 (H5N1) or A/Anhui/1/2013 (H7N9) virus-infected cells, guinea pig antibodies against H5N1 viral glycoproteins were used. Antibodies were prepared by immunizing guinea pigs and rabbits for four times every 2 weeks with A/Indonesia/5/2005 vaccine antigen (2 + 6 in PR8) (4 µg of HA/immunization) mixed with AS03 adjuvant (GlaxoSmithKline, Rixenart, Belgium) according to the instructions by the manufacturer. Secondary antibodies were fluorescein isothiocyanate (FITC)-labeled goat anti-guinea pig antibodies. In infectivity experiments, NucBlue Fixed Cell Ready Probes reagent (Life Technologies, DAPI) was added to secondary antibody staining solutions. Incubation time in every staining was 30 min in 0.5% BSA in PBS at 37°C and coverslips were washed three times with 0.5% Tween 20 (VWR) in PBS in every step. Finally, cells were washed with water and mounted in 25% Mowiol (Polysciences) in a solution containing 25 mM Tris–HCl (pH 7.5), 50% glycerol, and 2.5% 1,4-diazabicyclo(2,2,2)octane. The cells were imaged with Leica TCS SPE confocal microscope with a 63 1.40-numerical-aperture (NA) oil objective maintaining the same image acquisition settings for all acquired images. In the infectivity experiments, the cells were imaged with a Zeiss Stallion fluorescence microscope with a Hamamatsu ORCA-Flash 4.0 LT sCMOS camera and a 20 0.4-NA objective by using Slidebook 6 software (Intelligent Imaging Innovations). In the productivity assay, cells were calculated with 20 0.6-NA air objective with Leica TCS SPE confocal microscope.

### Flow Cytometry

For determining the infectivity of viruses in moDCs, the cells were differentiated on 6-well plates. Cells were infected with different virus dilutions and times as indicated in the figures. After different times of infection, cells from four different blood donors were harvested and handled separately. Cells were fixed with 4% PFA for 30 min, permeabilized with 0.1% Triton X-100 for 5 min, and stored with 0.5% BSA in PBS. A/Beijing/353/89 (H3N2) virus-infected cells were stained with rabbit antibodies against H3N2 glycoproteins (23). To visualize A/Vietnam/1194/2004 (H5N1) or A/Anhui/1/2013 (H7N9) virus-infected cells, antibodies against A/Indonesia/5/2005 (H5N1) vaccine antigen were used (see above). The secondary antibody was FITC-labeled goat anti-rabbit IgG (H + L) (Invitrogen, #F2765). In all antibody stainings, the cells were stained at RT for 1 h and washed twice with 0.5% BSA in PBS. The samples were analyzed with a FACSCanto II (BD) device using FACSDiva software.

### Western Blotting

For protein analysis, Mɸs or moDCs from four blood donors were harvested and pooled. We pooled the donors to be able to analyze higher amount of donors to get a more global and general view of the protein expression changes. Cell pellets were lysed with passive lysis buffer (Promega) containing 1 mM Na_3_VO_4_. Total cellular proteins were boiled in a Laemmli sample buffer, proteins were separated in SDS-PAGE gels and transferred onto Hybond-P polyvinylidene difluoride membranes (Amersham Biosciences). The membranes were blocked with 5% milk protein (Valio Co., Helsinki) in PBS. Anti-influenza A virus NP and M1 and MxA protein-specific antibodies were prepared as described previously (12, 24). Monoclonal anti-influenza A virus H7 HA antibody was obtained through BEI Resources, NIAID, NIH and anti-H3N2 glycoprotein antibodies and anti H5N1 virus antibodies were produced as described above. Antibody stainings for phosphorylated form of IRF3 (P-IRF3), P-STAT2, STAT2, and GAPDH proteins (Cell Signaling Technology) were performed according to the manufacturer’s instructions. For secondary antibodies, anti-rabbit or anti-mouse HRP-conjugated antibodies (Dako) were used. Protein bands were visualized on HyperMax films using a Pierce^®^ ECL Western Blotting substrate (Thermo Fisher Scientific).

### qRT-PCR

Infected Mɸs or moDCs from four blood donors were pooled (25), and total cellular RNA was isolated using the RNEasy Mini kit (Qiagen) and DNase digestion was performed with RNasefree DNase kit (Qiagen). 0.5 µg of total cellular RNA was transcribed to cDNA using TaqMan Reverse Transcriptase kit (Applied Biosystems) with random hexamers as primers. cDNAs were amplified by PCR using TaqMan Universal PCR Mastermix and Gene Expression Assays for IFN-λ1 (Hs00601677_g1), IFN-α1 (Hs00256882_s1), IFN-β1 (Hs00277188_s1), TNF-α (Hs01113624_g1), IL-1β (Hs00174097_m1), CXCL10 (Hs00171042_m1), CCL5 (Hs00174575_m1) (Applied Biosystems) or with primers and probes for influenza A virus M1 (12). The data were normalized to 18S rRNA with TaqMan Endogenous Control kit (Applied Biosystems). The relative gene expression in relation to an unstimulated sample was calculated with the ΔΔCT method according to instructions provided by Applied Biosystems.

To quantify viral RNA from the supernatant samples, moDCs and Mɸs were infected as described above and the supernatant samples were collected at 1 and 24 h after the infection. RNA was isolated using RNEasy Mini kit (Qiagen) with Qiacube (Qiagen). cDNA synthesis was done with RevertAid H Minus Reverse Transcriptase (Thermo Fisher Scientific) kit according to the manufacturer’s instructions with RiboLock RNase inhibitor (Thermo Fisher Scientific) and random hexamers (Roche) as primers. qRT-PCR was performed using QIAGEN^®^ QuantiTect™ Multiplex PCR NoRox Kit (Qiagen) with the same influenza A virus M1 primer–probe pair as above. The relative viral gene expression in relation to a 1-h sample was calculated with the ΔCT method according to the instructions provided by Applied Biosystems.

### Quantitation of Propagation-Competent Virus Particles

Macrophages were grown on glass coverslips and infected with MOI of 0.01. After 1 h incubation, virus dilution was removed, and cells were washed with PBS. Cells were overlaid with 1.2% Avicel in E-MEM (containing 2 µg/ml TPCK-trypsin, 0.3% BSA, 60 µg/ml streptomycin, 2 mM l-glutamine, and 20 mM HEPES) and incubated for 15 h. After incubation, the cells were washed two times with PBS, fixed with 4% PFA for 30 min, washed with PBS, permeabilized with 0.1% triton X-100 for 5 min, and stained with antibodies of different specificity (above-mentioned) followed by immunofluorescence analysis [adapted from Ref. (18)]. The percentage of productive vs. total infection events was determined by eye using confocal microscope with 20× objective and cells were imaged with 40× objective (Figures 7A,B).

### Statistical Analysis

The data was analyzed with the Student’s *t*-test to determine the statistical significance of differences between H5N1 virus and H3N2 or H7N9 viruses. Values of *p* < 0.05 were considered significant.

## Results

### Highly Pathogenic H5N1 Virus Has an Ability to Efficiently Spread in Primary Human Immune Cell Cultures

**Figure 1:**
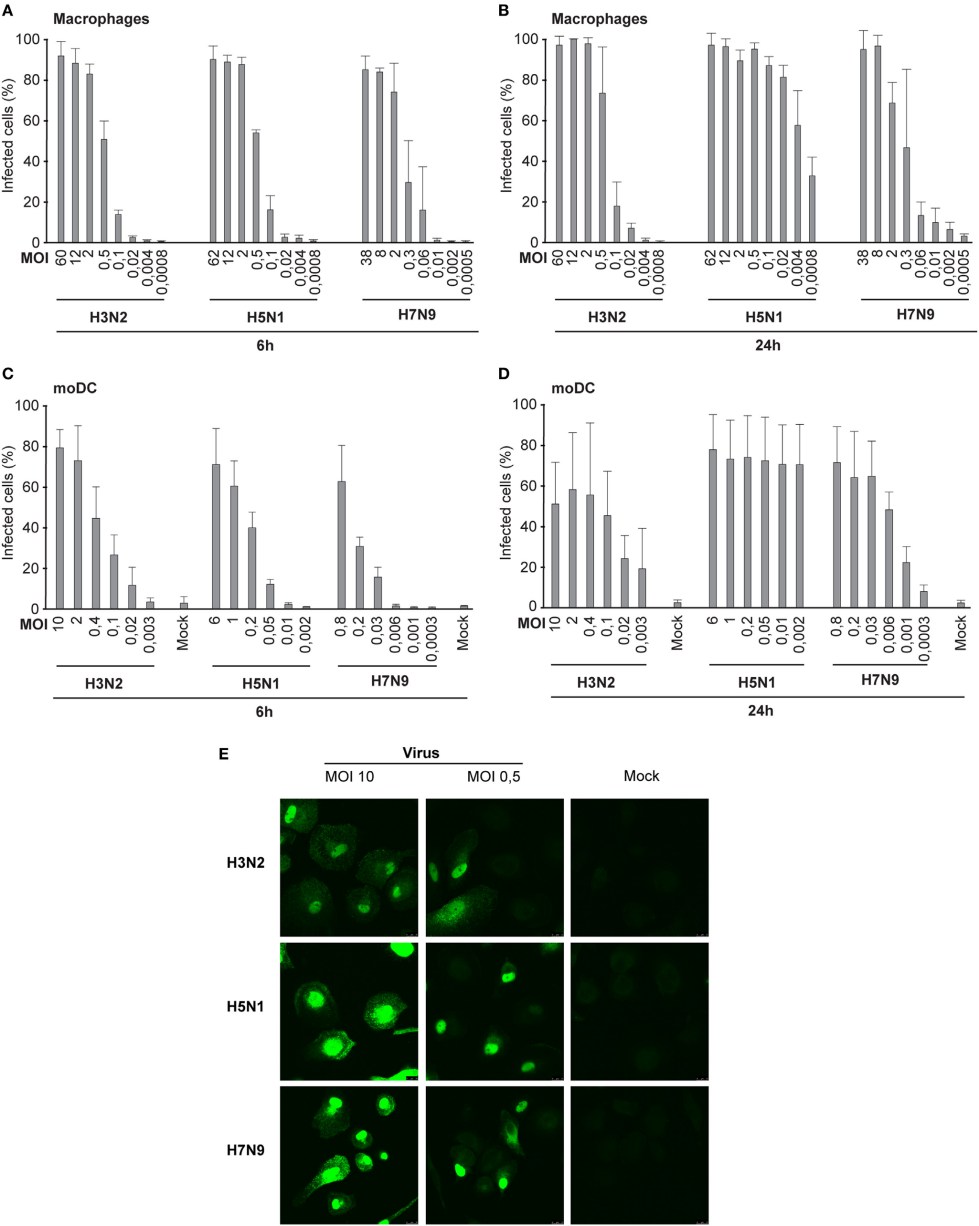
Infectivity of the H3N2, H5N1, and H7N9 influenza A viruses in human immune cells. **(A)** Human monocyte-derived macrophages (Mɸs) were infected with different doses of a seasonal influenza virus A/Beijing/353/89 (H3N2) or human isolates of avian influenza viruses A/Vietnam/1194/2004 (H5N1) or A/Anhui/1/2013 (H7N9) **(A)** for 6 h in the presence of oseltamivir carboxylate to prevent the spread of viruses, or **(B)** for 24 h in the absence of oseltamivir carboxylate. Infected Mɸs were detected with virus protein-specific antibodies by immunofluorescence microscopy. Results represent the mean values of three **(A)** or four **(B)** donors. From each donor at least 200 cells (average ca. 330) **(A)** or 300 cells (average ca. 840) **(B)** were counted. Human dendritic cells [monocyte-derived DCs (moDCs)] were infected with different virus doses **(C)** for 6 h in the presence of oseltamivir carboxylate, or **(D)** for 24 h in the absence of oseltamivir carboxylate. The proportion of infected moDCs was determined after staining the cells with antibodies against respective viral proteins for flow cytometry. From each donor, 10,000 events were analyzed. The mock sample (uninfected cells) was used to separate the uninfected and infected cells. Results are the mean values of four donors **(C,D)** analyzed separately. The infective particles per ml (FFU/ml) were calculated for Mɸs and moDCs separately, and multiplicity of infection (MOI) values was determined afterward. The MOI values in each experiment were calculated based on FFU/ml values determined in panels **(A,C)**. **(E)** Representative images of Mɸs infected with H3N2, H5N1, or H7N9 viruses at MOI 10 or 0.5 stained with virus-specific antibodies and secondary fluorescein isothiocyanate-labeled antibodies.

To determine the infectivity of influenza A/Beijing/353/89 (H3N2), A/Vietnam/1194/2004 (H5N1), and A/Anhui/1/2013 (H7N9) viruses in human Mɸs and dendritic cells (moDCs), we infected the cells with different virus doses for 6 h. We determined the number of infective units [focus forming units per milliliter (FFU/ml), Table 1] in virus stocks for both cell models separately from the dilution where approximately 50% of the cells were infected (Figures 1A,C,E). We used the FFU/ml values to determine the actual MOI. In the single-cycle infection (6 h), the infectivity curves with different doses of H5N1, H7N9, or H3N2 viruses both in Mɸs (Figure 1A) and moDCs (Figure 1C) corresponded well to the determined FFU/ml values. However, during a multi-cycle infection (24 h), H5N1 virus was able to efficiently spread in the culture and the virus infected all cells even with the very low MOI value in the beginning of the infection (Figures 1B,D).

All virus stocks were propagated in low passages in chicken eggs and the stock virus infectivity titers in different cell types were assessed. To determine the titers of infective units in the virus stocks, we performed a plaque forming assay in MDCK cells. In these cells, the H5N1 strain from 2004 gave the lowest titers when compared with those of H7N9 and H3N2 viruses (Table 1). The reason for this could be that the H7N9 virus has a high affinity to both α-2,3- (avian type) and α-2,6-linked (human type) sialic acids whereas usually the avian type H5N1 viruses prefer the α-2,3-type receptor (26). However, in moDCs and Mɸs where both α-2,3- and α-2,6-linked sialic acids are expressed (14, 15), the infectivity titer of H5N1 stock was the highest when compared with H3N2 or H7N9 viruses. Moreover, it seems that human Mɸs are more permissive than moDCs to all of these viruses (Table 1).

**Figure 2:**
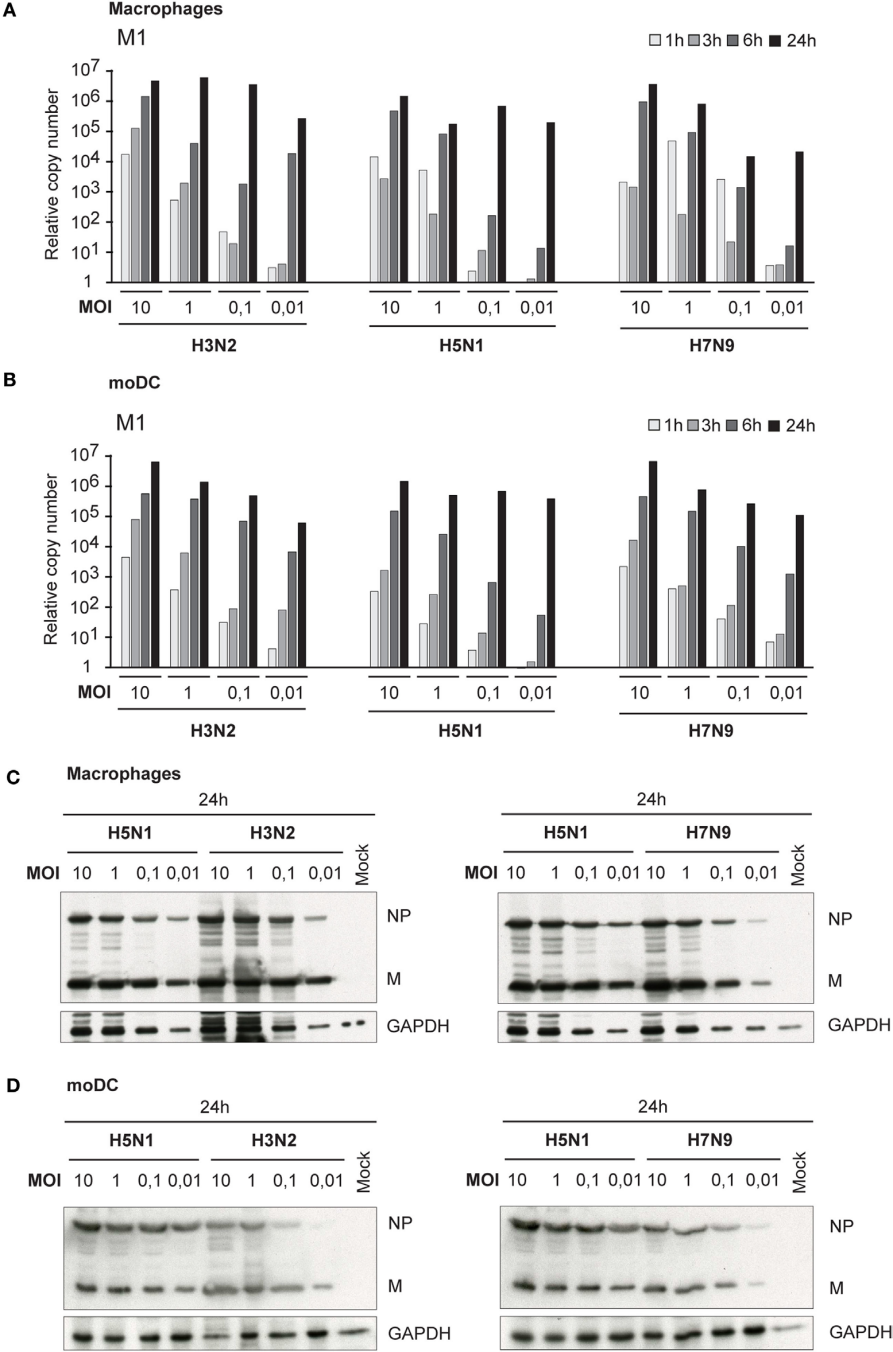
Viral RNA expression and protein synthesis in H3N2, H5N1, and H7N9 virus-infected monocyte-derived macrophages (Mɸs) and monocyte-derived DCs (moDCs). Mɸs and moDCs from four different blood donors were separately infected with A/Beijing/353/89 (H3N2), A/Vietnam/1194/2004 (H5N1), and A/Anhui/1/2013 (H7N9) influenza viruses at different multiplicity of infection (MOI) values for up to 24 h. **(A)** Mɸs and **(B)** moDCs were collected at 1, 3, 6, and 24 h after infection, cells from different donors were pooled and total cellular RNA was isolated for measuring of viral M1 RNA expression by qRT-PCR. The fold induction was calculated over mock sample. The data shown are representative of three independent experiments. **(C,D)** Cellular protein lysates from **(C)** Mɸs or **(D)** moDCs were collected at 24 h after infection, samples from different donors were pooled and viral NP and M1 protein expression was analyzed using Western blotting. GAPDH levels were analyzed to control the equal loading of the samples. A representative experiment out of two (Mɸs) or three (moDC) is shown.

Besides primary infectivity in Mɸs and moDCs, we investigated the replication of these viruses in Mɸs and moDCs by analyzing the expression of viral M1 RNA with qRT-PCR. During the first 6 h of infection, we did not observe any clear differences in M1 gene expression between H3N2, H5N1, or H7N9 viruses, which indicate that the rate of viral replication is similar between the viruses during the primary infection. In the secondary infection phase (24 h time point) in H5N1-infected cells, the expression of M1 gene reached the same submaximal levels regardless of the MOI values used (0.01–10) (Figures 2A,B). In H3N2 and H7N9 virus-infected cells, instead, there was a clear dose-dependent expression of viral M1 RNA in different time points (Figures 2A,B). In addition, there was a clear difference in the production of viral NP and M proteins between H5N1 and H7N9 or H3N2 virus-infected cells. In H5N1 virus-infected cells viral protein expression was high, independent of the MOI value used in the beginning of the infection, while in H3N2 or H7N9-infected cells viral protein levels depended on the virus dose at the beginning of the infection (Figures 2C,D).

### The HA of H5N1 Virus Is Efficiently Cleaved in Human Immune Cells

**Figure 3:**
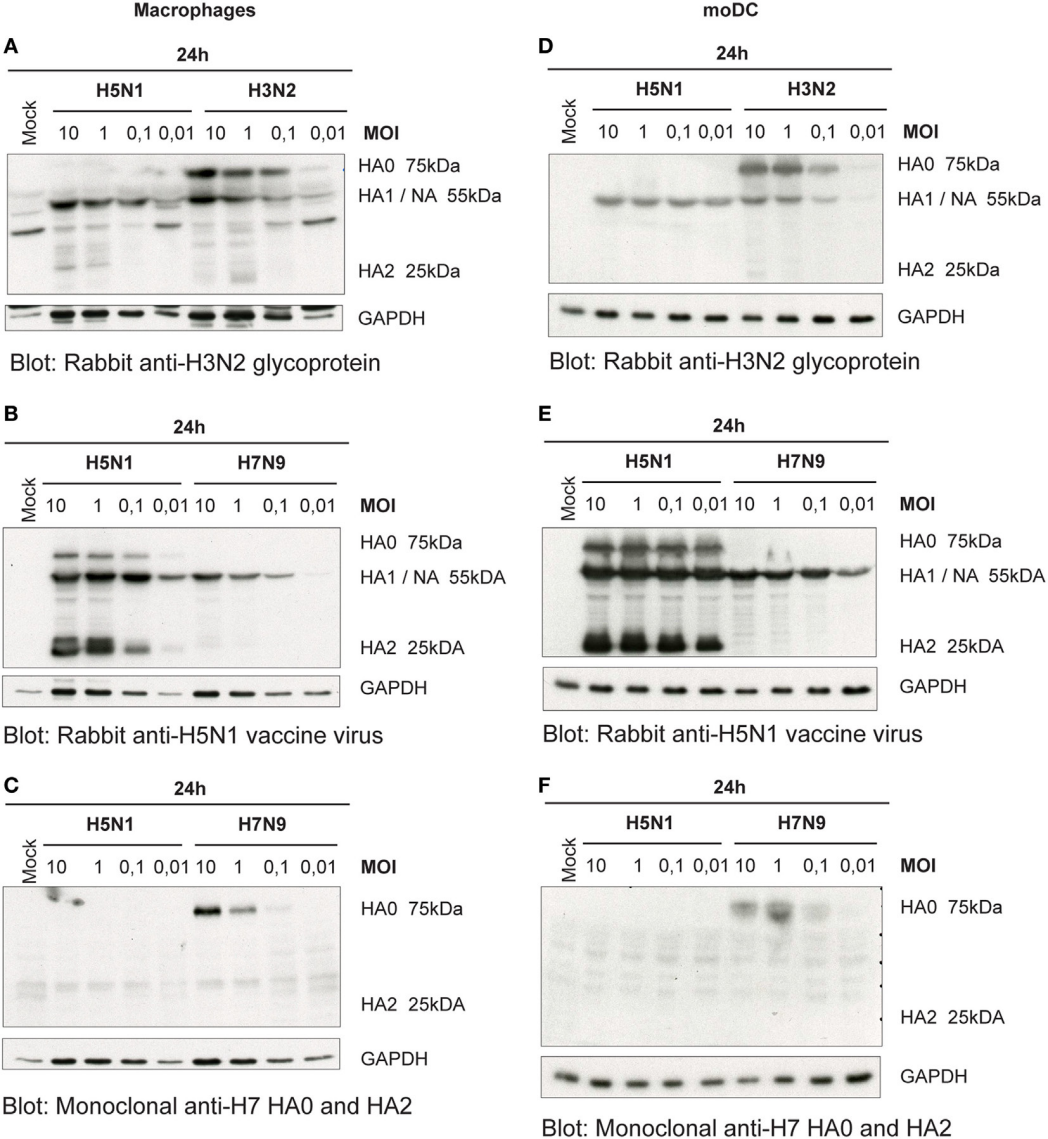
Cleavage of the hemagglutinin (HA) protein of the influenza viruses in infected **(A–C)** monocyte-derived macrophages (Mɸs) and **(D–F)** monocyte-derived DCs (moDCs). Mɸs and moDCs from four blood donor were infected with A/Beijing/353/89 (H3N2), A/Vietnam/1194/2004 (H5N1), and A/Anhui/1/2013 (H7N9) influenza viruses at indicated multiplicity of infection (MOI) values for 24 h. Cellular lysates for protein samples were collected, the samples of different donors were pooled, and HA proteins were analyzed by Western blotting. The membranes were stained **(A,D)** with antibodies against glycoproteins of H3N2 virus to detect the HA0, HA1, and HA2 of the H3N2 virus, **(B,E)** against the glycoproteins of H5N1 virus to detect the HA0, HA1 and HA2 of the H5N1 virus, or **(C,F)** against the HA of H7 virus to detect HA0 and HA2 of the H7N9 virus. In panels **(A,B)**, HA1 and NA co-migrate and thus the heterologous signal is due to cross-reactivity of the NA protein. The levels of GAPDH were used as an internal loading control. A representative experiment out of two is shown.

For the newly produced viruses to be infective, their HA precursor (HA0) has to be cleaved to HA1 and HA2 subunits by cellular proteases. Thus, we next analyzed how efficiently the HA molecule of H5N1, H7N9, and H3N2 viruses is cleaved in human immune cells. As visualized by immunoblotting in H3N2 virus-infected Mɸs, there was only some or none of the HA2 subunit detectable (Figure 3A). The A/Vietnam/1194/04 (H5N1) strain has multiple arginine and lysine residues at the HA1–HA2 cleavage site as a marker for a highly pathogenic form of an avian influenza virus. This multi-basic cleavage site can be cleaved by ubiquitous cellular proteases and, indeed, we noticed that in H5N1-infected human Mɸs a great amount of the HA0 protein produced at 24 h even with very low MOI was efficiently cleaved to HA1 and HA2 subunits (Figure 3B). However, like in H3N2 infection, in H7N9 virus-infected Mɸs, there was only some or none of the HA2 subunit detectable (Figure 3C). The HA of the different viruses was cleaved in similar fashion also in moDCs (Figures 3D–F).

### Addition of Trypsin Does Not Increase the Infectivity of H3N2 or H7N9 Viruses to the Levels Seen With the H5N1 Virus

**Figure 4:**
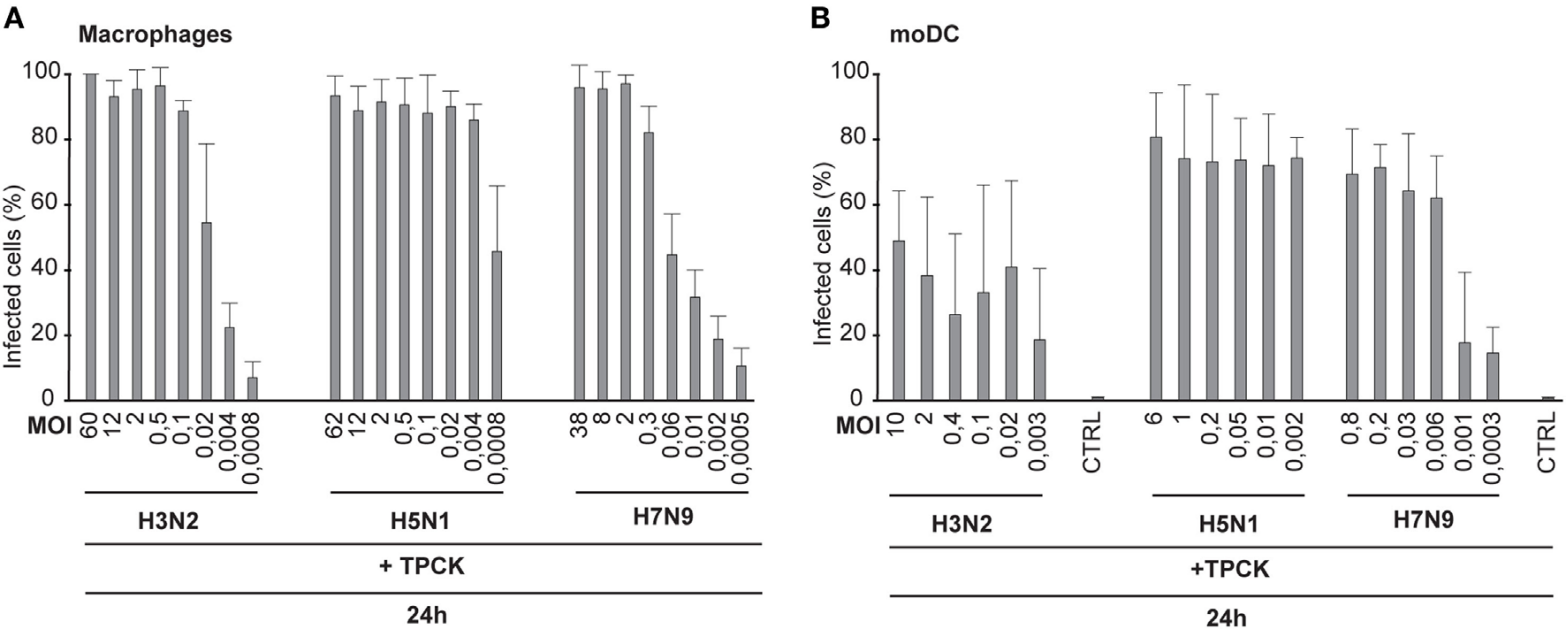
Infectivity of the H3N2, H5N1, and H7N9 viruses in the presence of trypsin. Monocyte-derived macrophages (Mɸs) **(A)** or monocyte-derived DCs (moDCs) **(B)** were infected with externally added TPCK-trypsin with A/Beijing/353/89 (H3N2), A/Vietnam/1194/2004 (H5N1), and A/Anhui/1/2013 (H7N9) influenza viruses with indicated multiplicity of infection (MOI) values and the samples were collected at 24 h after infection. **(A)** The proportion of infected Mɸs was analyzed with immunofluorescence microscopy by staining the cells with antibody against respective virus proteins. From each donor at least 300 cells (average ca. 550) were counted. **(B)** moDCs were stained with antibody against virus proteins and infected cells were analyzed with flow cytometry. From each donor 10,000 events were analyzed.

Next we investigated whether the trypsin-induced cleavage of HA contributes to influenza virus spread in immune cells. Human Mɸs and moDCs were infected with H3N2, H5N1, and H7N9 viruses in the presence of TPCK-trypsin to induce the cleavage of HA0 into HA1 and HA2 subunits and the number of virus-infected cells was analyzed by immunofluorescence or flow cytometry, respectively. We noticed that trypsin increased the infectivity of the H3N2 and H7N9 viruses to some extent at the 24-h time point (compared Figures 4A,B with Figures 1B,D) but yet the infectivity of H3N2 and H7N9 remained at a much lower level when compared with that of the H5N1 virus. In Mɸs, even at a MOI of 0.004 nearly 100% of cells were infected by the H5N1 virus while in H3N2 or H7N9 infection less than 40% of the cells were infected (Figure 4A). In moDCs, the difference was equally clear and in H3N2 or H7N9 infection with the lowest MOI values only ca. 20% of the cells were infected while with similar H5N1 virus dose most cells were infected (Figure 4B). Unexpectedly, the infectivity of H3N2 or H7N9 virus, even with added trypsin, did not become as effective as was seen with H5N1 virus. This suggests that H5N1 can spread in human immune cells extremely efficiently and this spread is not solely dependent on the effective cleavage of HA.

### H5N1 Virus Is Able to Spread in Immune Cell Starting From Only a Few Infectious Virus Particles

**Figure 5:**
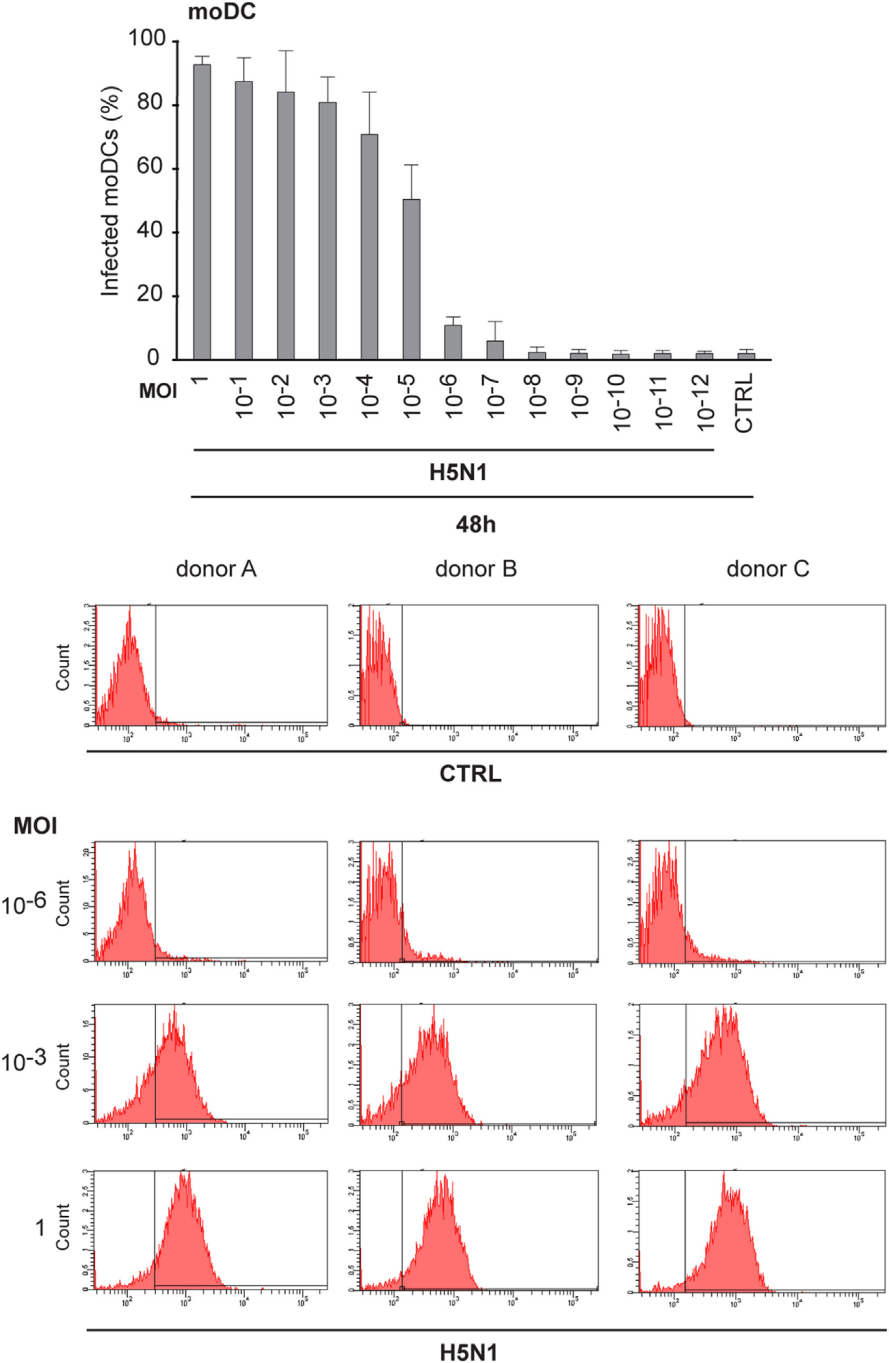
Infectivity of H5N1 virus with serial dilutions in monocyte-derived DCs (moDCs). To titrate the infectivity and the spread of infection, A/Vietnam/1194/2004 (H5N1) virus stock was diluted in 1:10 series from multiplicity of infection (MOI) 1 to MOI 10–12 and moDCs from 3 different donors were infected for 48 h. Cells were collected and the percentage of infected cells was analyzed with flow cytometry. From each donor 10,000 events were analyzed.

Next, we analyzed how low amount of the H5N1 virus was sufficient to infect the whole immune cell culture. To do this, we made dilutions series of the stock virus and infected moDCs with MOI values ranging from 1 to 10^−12^ for 48 h. Interestingly, most moDCs were infected even at a MOI 10^−5^ of H5N1 virus and the infectivity decreased to background levels at a MOI of 10^−8^, where there should not be any viral particles left (Figure 5). The findings suggest that the H5N1 virus is able to start a productive infection and spread eventually to the whole cell culture even from one infectious virus particle.

### An Efficient Spread in Human Immune Cells and Efficient HA Cleavage Are General Features of HPAI H5N1 Viruses

**Figure 6:**
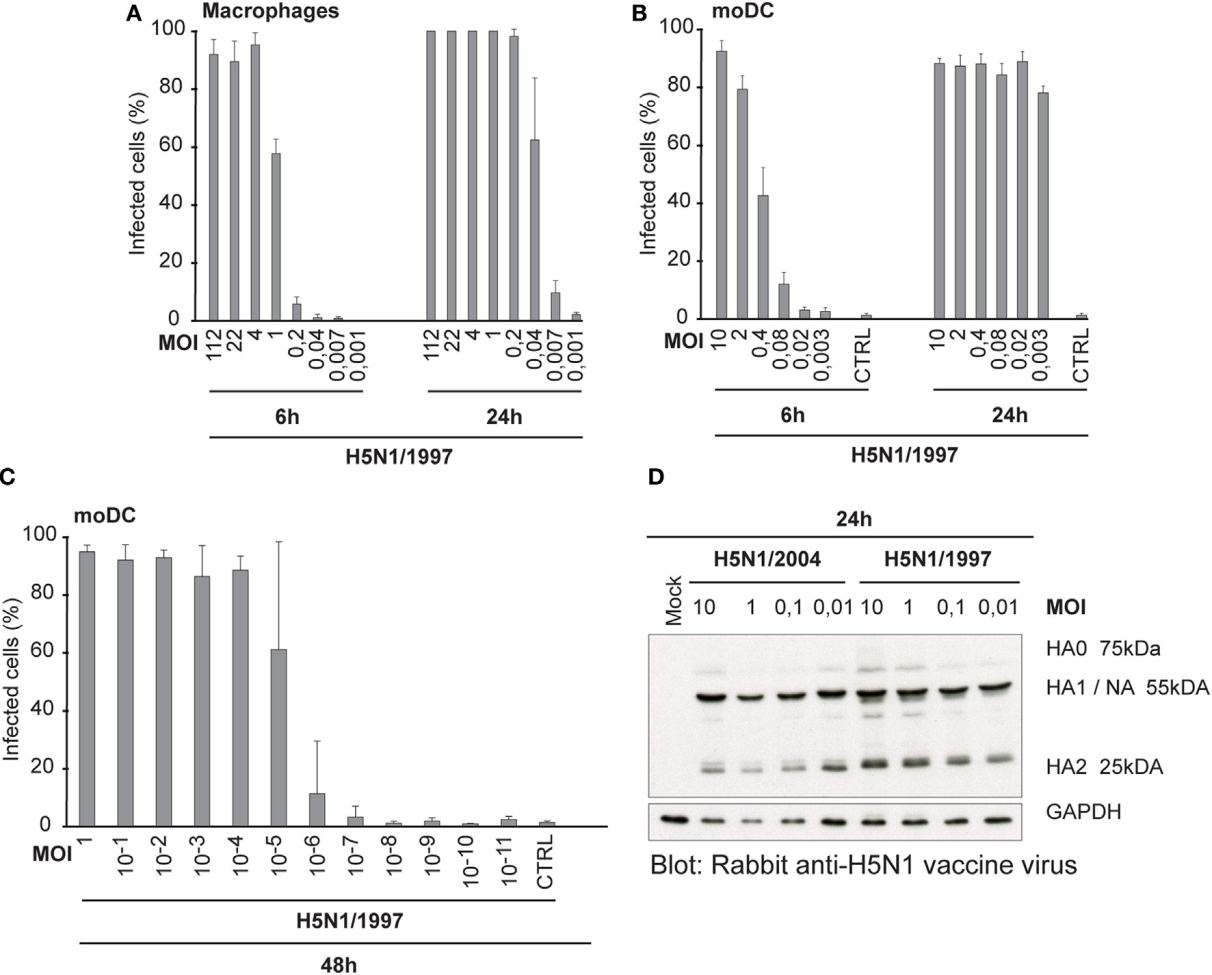
Infectivity and the cleavage of the HA protein of A/Hong Kong/156/1997 (H5N1) virus. **(A)** Monocyte-derived macrophages (Mɸs) or **(B)** monocyte-derived DCs (moDCs) were infected with different doses of a human isolate of avian influenza A/Hong Kong/156/1997 (H5N1) virus for 6 or 24 h. Results are the mean values from four donors infected and analyzed separately. Infected cells were detected with antibodies against H5N1 vaccine antigens **(A)** using immunofluorescence microscopy or **(B)** with flow cytometry. From each donor, at least 200 cells (average ca. 420) were counted **(A)** or 10,000 events were analyzed **(B)**. The titers for infective particles per ml (FFU/ml) and representative multiplicity of infection (MOI) values were calculated for Mɸs and moDCs. **(C)** moDCs were infected with H5N1/1997 virus at different MOI values for 48 h. The cells were stained with virus-specific antibodies and analyzed with flow cytometry. From each donor, 10,000 events were analyzed. Results are the mean values of four donors analyzed separately. **(D)** moDCs from four donors were infected with indicated MOI values of H5N1/1997 virus for 24 h with or without added TPCK-trypsin and for protein samples cellular lysates from different donors were pooled. Cleavage of HA protein was detected by Western blotting with antibodies against the glycoproteins of H5N1 virus to detect the HA0, HA1, and HA2 of the H5N1 virus.

Our next question was, whether the ability to spread in human immune cells is only associated with this specific HPAI H5N1 strain A/Vietnam/1194/04 or whether it is a more universal feature of all highly pathogenic avian-origin H5N1 viruses. Thus, we included another highly pathogenic H5N1 strain into the study. This A/Hong Kong/156/1997 H5N1 virus gave almost an equally high titer in MDCK cells than the H7N9 virus, whereas the other H5N1/2004 strain gave the lowest PFU titer (Table 1). It may be that the H5N1/1997 virus is already adapted to the mammalian MDCK cells, because H5N1/1997 virus has been cultivated at least four passages in MDCK cells before the propagation in chicken eggs. This likely explains the high PFU/ml titer of H5N1/1997 virus. The infectivity of A/Hong Kong/156/1997 (H5N1) virus was also determined in Mɸs and moDCs at 6 and 24 h by immunofluorescence microscopy and flow cytometry, respectively (Figures 6A,B), and calculated the FFU/ml values (Table 1) for virus stocks as was done for H3N2, H5N1/2004, and H7N9 viruses (Figures 1A,C). In multi-cyclic infection at 24 h, H5N1/1997 virus was spreading efficiently in Mɸs and moDCs (Figures 6A,B) in a similar fashion as the H5N1/2004 virus (Figures 1B,D). Infection with H5N1/1997 virus at MOI value of 10^−5^ reached over 60% infection rate in moDCs at 48 h. In addition, 10% of the cells got infected from the virus dose of MOI 10^−6^ where theoretically only one infectious virus particle was added into the culture (Figure 6C). Furthermore, the HA of the H5N1/1997 virus was efficiently cleaved from HA0 to HA1 and HA2 subunits as was also the case with the HA of the H5N1/2004 virus (Figure 6D). These data show that the H5N1/1997 virus has a similar ability to spread in human Mɸs and moDCs as the H5N1/2004 virus and the cleavage of HA is extremely efficient in these cell types suggesting that these features are general to HPAI H5N1 viruses.

### Primary Infective Units of H5N1 Virus Are More Often Propagation-Competent and Productive Than Those of H3N2 or H7N9 Viruses in Human Immune Cells

**Figure 7:**
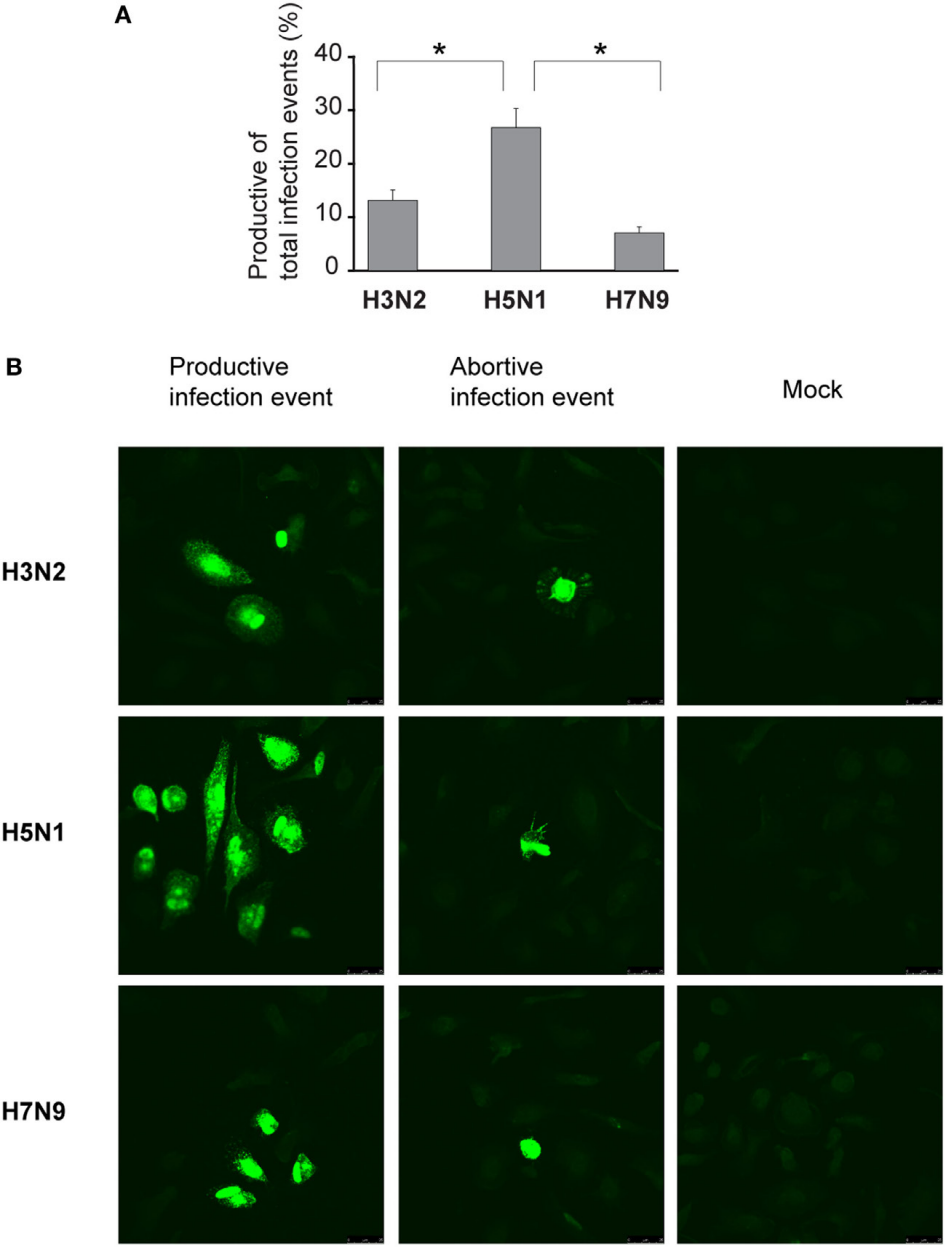
Proportion of the propagation-competent virus particles in virus stocks. Monocyte-derived macrophages (Mɸs) were grown on glass coverslips, infected with A/Beijing/353/89 (H3N2), A/Vietnam/1194/2004 (H5N1), or A/Anhui/1/2013 (H7N9) influenza viruses at multiplicity of infection (MOI) of 0.01. After 1 h incubation, cells were washed and overlaid with 1.2% Avicel in E-MEM with added trypsin. After 15 h incubation, cells were fixed, permeabilized, and stained for viral protein expression. **(A)** Productive and abortive infection events were calculated. The results represent the mean values + SDs of the means from the cells of four different donors. From each donor, four coverslips with 80 to 500 infection events were counted. The statistical significance of differences between H5N1 and H3N2 or H7N9 was determined by Student’s *t*-test, *<0.05. **(B)** Representative immunofluorescence images of infection events for each virus. Infection events were considered productive if three or more adjacent cells were infected. If two or less adjacent cells were infected, the infection event was considered abortive. Immunofluorescence images from one representative donor are shown.

**Figure 8:**
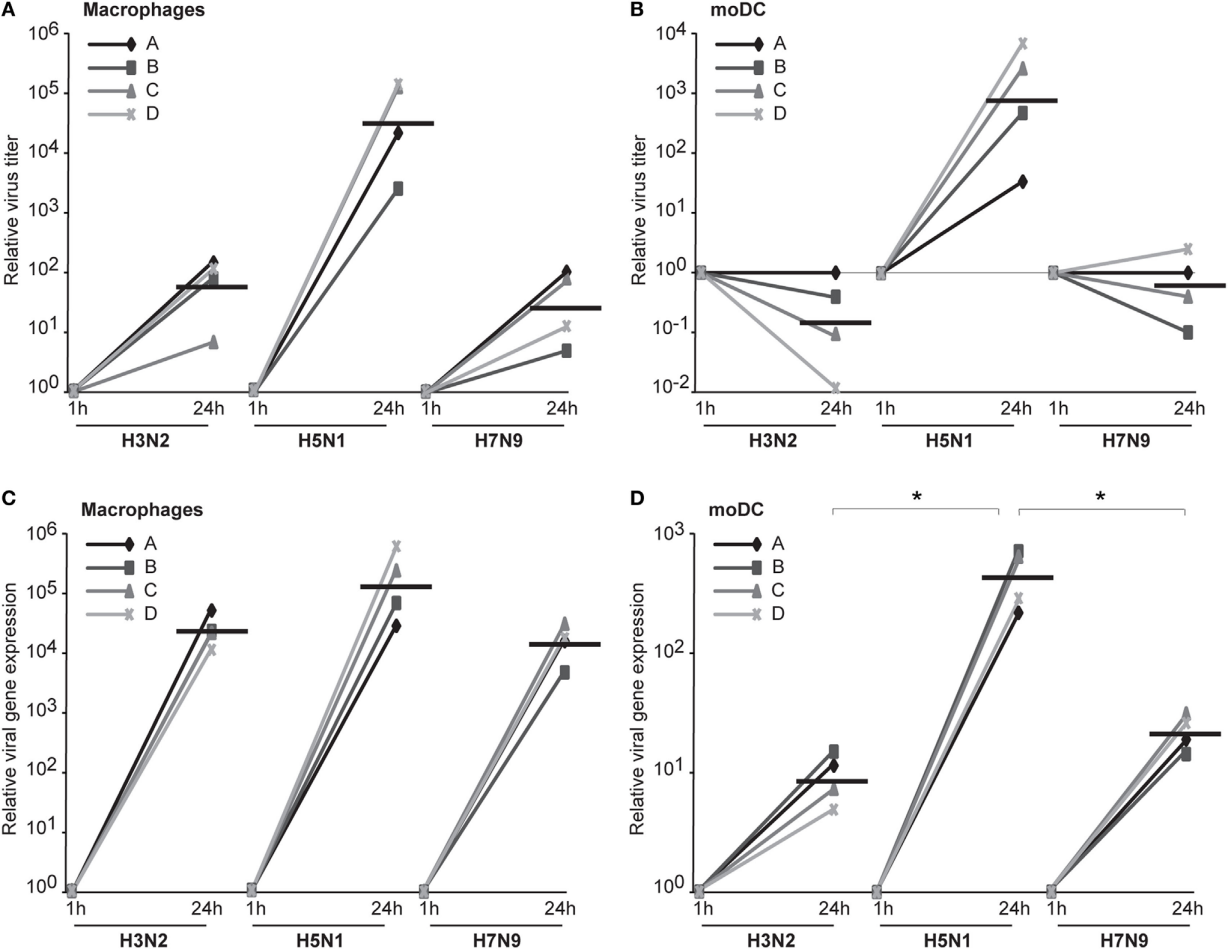
The productivity of H3N2, H5N1, and H7N9 infections in human innate immune cells. Monocyte-derived macrophages (Mɸs) or monocyte-derived DCs (moDCs) from four different donors were infected with A/Beijing/353/89 (H3N2), A/Vietnam/1194/2004 (H5N1), or A/Anhui/1/2013 (H7N9) influenza viruses at multiplicity of infection (MOI) of 0.01 and after 1 and 24 h of infection the supernatant samples were collected. **(A,B)** The infective viral titers produced from Mɸs **(A)** or moDCs **(B)** were determined by plaque assay in Madin–Darby Canine Kidney cells. The relative virus titers were calculated over 1 h samples. The horizontal lines represent the geometric means of the results from four blood donors. **(C,D)** The RNA was isolated from the supernatant samples from Mɸs **(C)** and moDCs **(D)** and the viral RNA levels were detected by qRT-PCR. The relative viral RNA expressions were calculated over 1 h samples with ΔCT method. The horizontal lines represent the geometric means from different donors. The statistical significance of differences between H5N1 and H3N2 or H7N9 was determined by Student’s *t*-test, *<0.05.

We assumed that one reason for the efficient spread of H5N1 virus may be that H5N1 virus particles are more often packed with a complete set of gene segments and therefore these virus particles are propagation-competent and infection events are productive. To investigate this possibility, we set up the propagation-competent virus particle test (18). Human primary Mɸs were plated on coverslips followed by infection with H3N2, H5N1 from 2004 and H7N9 viruses at MOI of 0.01, overlaid with Avicel cellulose, incubated 15 h, fixed, and the infected cells stained for fluorescent microscopic analysis. Infection events were considered productive when three or more adjacent cells were infected. Our results show that significantly more infection events were productive with H5N1 virus, with almost 30% of the total infection events being productive, when compared with the 12 and 8% of events with H3N2 and H7N9 viruses, respectively (Figure 7A). We also noticed that the clusters of H5N1 virus-infected cells were larger and consisted of even 10 cells while those with H7N9 or H3N2 viruses were smaller and consisted of only a few cells (Figure 7B). Next, we analyzed whether the H3N2, H5N1, or H7N9 virus infections were productive by propagating new infectious virions in human Mɸs and moDC. For that, we performed plaque assays from the supernatant samples collected at 1 or 24 h after the infection. We noticed that the amount of infectious virus in the Mɸ cell culture supernatant was increased with all the studied viruses but H5N1 was superior, producing approximately 1,000-fold higher virus titers (Figure 8A). In moDCs, the virus titers increased even 10,000-fold in H5N1 virus infection after 24 h (Figure 8B). In contrast to the H5N1 virus infection, in infections with H3N2 or H7N9 viruses, the virus titers had a tendency to decrease (Figure 8B). In addition to analyzing the amount of secreted infectious virus, we investigated the viral RNA levels at 1 and 24 h in the Mɸs and moDCs supernatants of H3N2, H5N1, and H7N9 virus-infected cells. In H5N1 virus-infected Mɸs and moDC supernatants, total viral RNA levels increased over 100,000- and 100-fold, respectively, whereas in H3N2 or H7N9 virus-infected cells, viral RNA levels increased ca 10,000-fold in Mɸs and 10-fold in moDCs (Figures 8C,D). The data indicate that the H5N1 virus spreads more efficiently than H3N2 or H7N9 viruses in human immune cell cultures leading to efficient secretion of newly produced infectious H5N1 viruses.

### H5N1 Virus Infection Is Able to Spread in Human Immune Cell Cultures Irrespective of Induced IFN Responses

**Figure 9:**
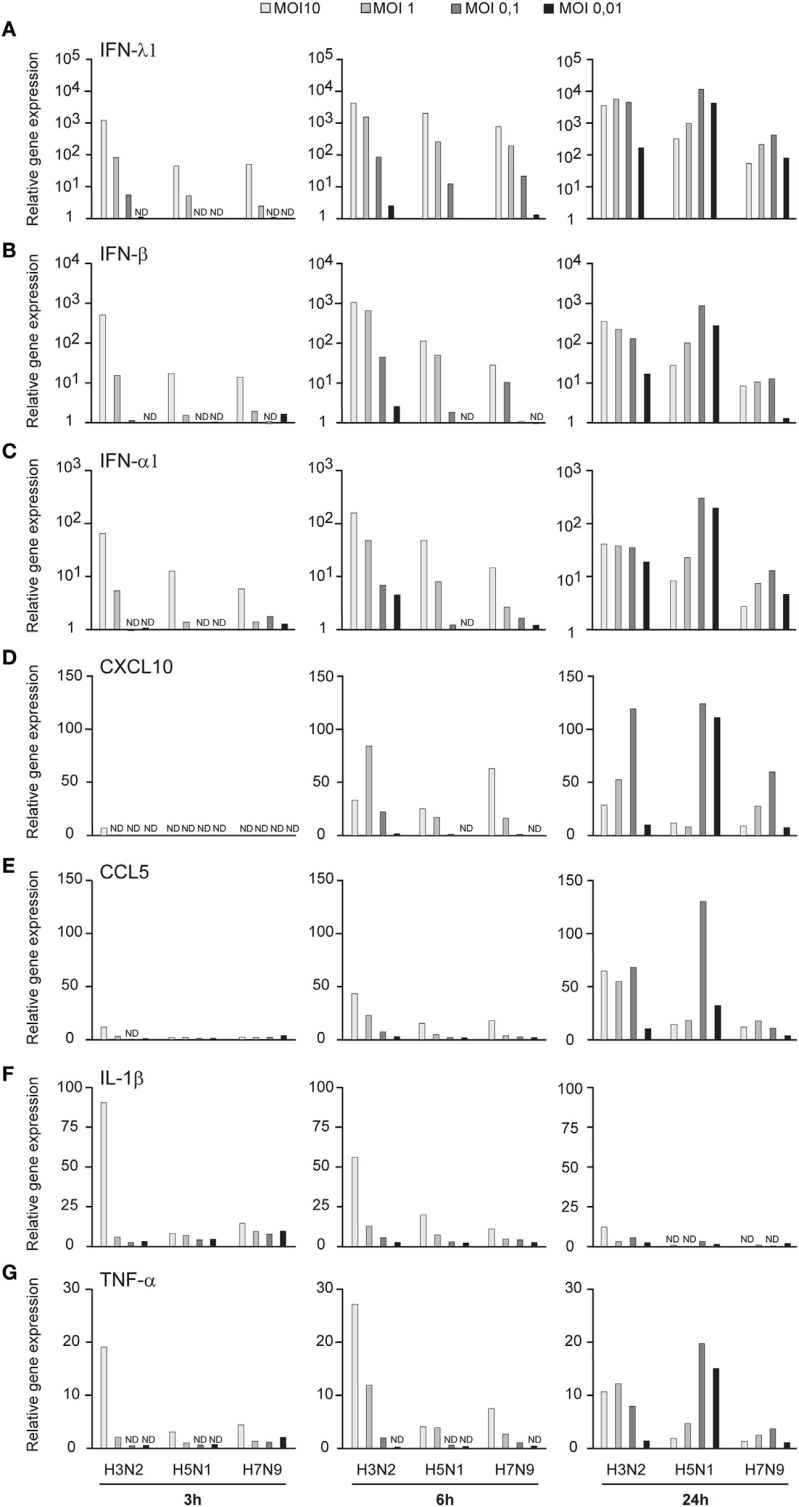
Innate immune responses in H3N2, H5N1, and H7N9 influenza virus-infected human monocyte-derived macrophages (Mɸs). Mɸs were infected with A/Beijing/353/89 (H3N2), A/Vietnam/1194/2004 (H5N1), or A/Anhui/1/2013 (H7N9) influenza viruses at multiplicity of infection (MOI) values of 10 to 0.01 for 24 h. **(A)** Cells from four donors were collected at different time points after infection as indicated in the figure, cells from different donors were pooled and total cellular RNA was isolated. **(A)** IFN-λ1, **(B)** IFN-β, **(C)** IFN-α1, **(D)** CXCL10, **(E)** CCL5, **(F)** IL-1β, and **(G)** TNF-α gene expression was analyzed by qRT-PCR. Gene expression < 1 is defined as not detected (ND). A representative experiment out of two is shown.

**Figure 10:**
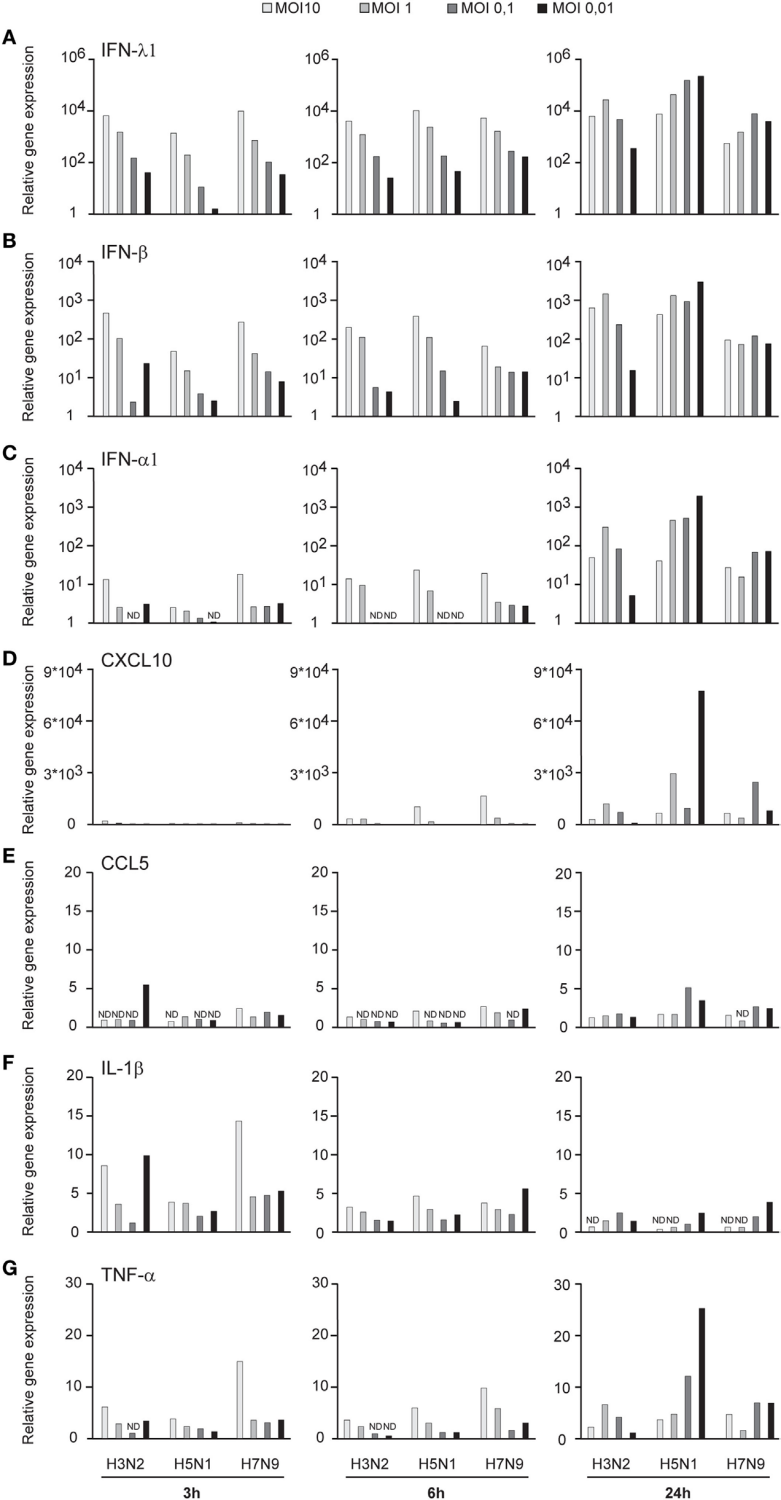
Innate immune responses in H3N2, H5N1, and H7N9 influenza virus-infected human monocyte-derived DCs (moDCs). moDCs were infected with A/Beijing/353/89 (H3N2), A/Vietnam/1194/2004 (H5N1), or A/Anhui/1/2013 (H7N9) influenza viruses at multiplicity of infection (MOI) values of 10 to 0.01 for 24 h. **(A)** Cells from four donors were collected at different time points after infection as indicated in the figure, cells from different donors were pooled and total cellular RNA was isolated. **(A)** IFN-λ1, **(B)** IFN-β, **(C)** IFN-α1, **(D)** CXCL10, **(E)** CCL5, **(F)** IL-1β, and **(G)** TNF-α gene expression was analyzed by qRT-PCR. Gene expression < 1 is defined as not detected (ND). A representative experiment out of three is shown.

**Figure 11:**
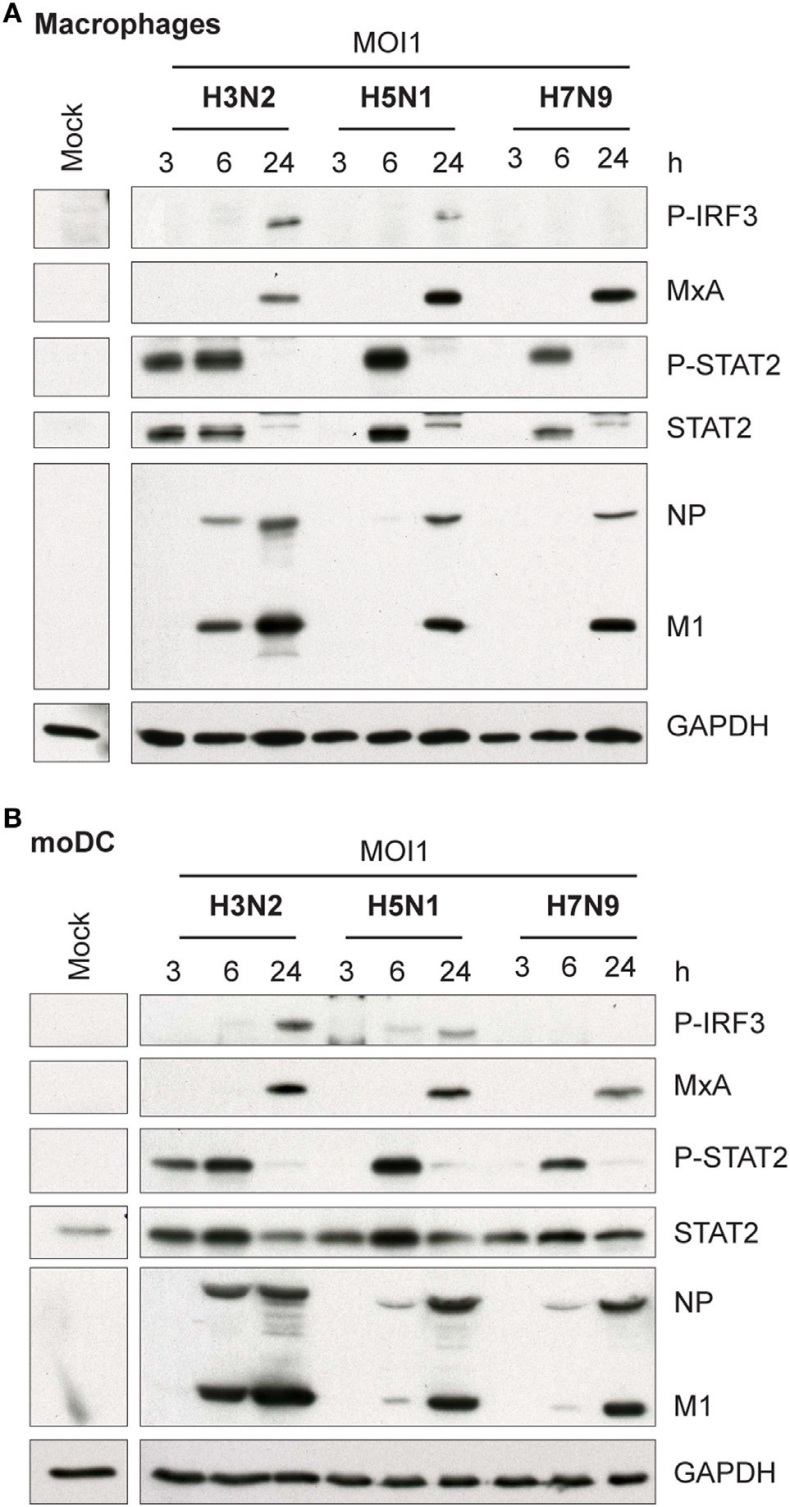
Activation of transcription factors and expression of antiviral MxA protein in H3N2, H5N1, and H7N9 influenza virus-infected human monocyte-derived macrophages (Mɸs) and monocyte-derived DCs (moDCs). **(A)** Mɸs and **(B)** moDCs were infected with A/Beijing/353/89 (H3N2), A/Vietnam/1194/2004 (H5N1), or A/Anhui/1/2013 (H7N9) influenza viruses at multiplicity of infection (MOI) values of 1. Cellular protein lysates were collected at 3, 6, and 24 h after infection, samples from four donors were pooled and P-IRF3, MxA, P-STAT2, STAT2, and viral NP and M1 protein expression was analyzed by Western blotting using specific antisera. GAPDH protein expression was analyzed to control equal loading of the samples. A representative experiment out of two (Mɸs) or three (moDCs) is shown.

Influenza virus infection induces type I and III IFN production in response to the recognition of viral RNAs by host receptors. We and others have previously shown that IFN-α can inhibit H3N2, H5N1, and H7N9 virus replication (12, 27). Here, we show that the H5N1/2004 avian influenza strain replicates and spreads efficiently in Mɸs and moDCs even at very low multiplicity infection (MOI 0.01) (Figures 2A,B), although the host cells respond to the infection with maximal cytokine gene expression (Figures 9 and 10). The H5N1/2004 virus induced strong IFN-λ1, IFN-α1, and IFN-β gene expression in Mɸs (Figures 9A–C). This suggests that the H5N1 virus is able to spread irrespective of the activation of antiviral immune responses since otherwise IFNs should inhibit virus replication. Lower virus doses in H3N2 or H7N9-infected moDCs led to clearly weaker IFN responses when compared with H5N1-infected cells at the 24 h time point (Figures 9A–C) suggesting that there are likely less infected cells that produce IFNs. To get further view of H5N1/2004 virus induced “cytokine storm,” we investigated the expression of chemokine CXCL10 and CCL5 and pro-inflammatory cytokine IL-1β and TNF-α genes in H3N2, H5N1/2004, or H7N9 virus-infected Mɸs (Figures 9D–G). Like IFN responses, with low MOI value, also CXCL10, CCL5, and TNF-α responses were higher in H5N1-infected Mɸs with low MOI value than with low MOI values in H3N2 or H7N9 virus infection. Induction of IL-1β gene expression is very low with H3N2, H5N1, and H7N9 viruses (Figure 9F) as we have also previously shown in moDCs (12). IFN-λ1, IFN-α1, IFN-β, CXCL10, CCL5, IL-1β, and TNF-α gene expression was also investigated in H3N2, H5N1, and H7N9 virus-infected moDCs (Figure 10). Data from moDCs correlate with the data from virus-infected Mɸs though CXCL10 mRNA induction was clearly higher in moDCs than in Mɸs, whereas CCL5 gene was expressed in higher levels in Mɸs. Thus, we next analyzed the expression of antiviral proteins in H3N2, H5N1, and H7N9-infected Mɸs (Figure 11A) and moDCs (Figure 11B). With all analyzed viruses, the expression of IFN-induced antiviral MxA protein was the strongest at the 24-h time point (Figures 10A,B). We also analyzed the expression of phosphorylated transcription factors IRF3 (P-IRF3) and STAT2 (P-STAT2) and noticed that the protein expression of these proteins was weaker in H7N9-infected cells compared with those seen in H5N1 or H3N2 infected cells (Figures 11A,B) which correlates well with the gene expression of IFNs (Figures 9 and 10) as well as with our previous study (12). The data show that in a multi-cyclic infection the H5N1 virus is able to induce very strong IFN gene expressions and antiviral MxA protein expression.

## Discussion

In 1997, a direct transmission of an avian influenza H5N1 virus from poultry to humans was documented for the first time (28). After that many subtypes of avian influenza A viruses have been reported to cause infections in humans, among them the H7N9 influenza type (4). High prevalence of influenza viruses in birds, the possibility of formation of new highly pathogenic reassortants and the ability of these viruses to spread to humans makes avian influenza A viruses of great global concern and emphasize the importance of virus–host cell interaction studies in human cell systems. In this study, we wanted to characterize different steps of H5N1 virus infection in human immune cells to reveal potential mechanisms explaining the severe clinical outcome of the H5N1 virus infection. In our previous study, we have shown that the avian H5N1 and H7N9 viruses can replicate in human moDCs (12). Here, we show that, unlike the H7N9 or seasonal influenza A viruses, the H5N1 virus is efficiently spreading in human immune cell cultures leading to a productive infection and robust IFN response. The remarkable ability of H5N1 virus to spread in human immune cells and likely other human cell types can be one explanation for the severe disease seen in humans with H5N1 virus infection. Here, we show that this ability is associated with at least two different HPAI H5N1 virus strains, and it may be a universal feature for all highly pathogenic avian-origin influenza A virus subtypes. There may be other viral or host factors regulating avian influenza pathogenicity in humans, since the LPAI H7N9 virus (LPAI in birds) has caused more human infections and deaths than the H5N1 subtype (4). In addition, there is now evidence that the H7N9 subtype has evolved to a HPAI H7N9 strain in birds, and this type of virus has already caused infections in humans (5).

Human infections with the HPAI H5N1 viruses are associated with a high viral load (29), and our data presented in this study confirm that the ability of the H5N1 virus to replicate and spread in immune cell culture is completely different from those of the LPAI H7N9 and seasonal H3N2 influenza A virus infections (Figures 1, 2 and 6A,B). The efficacy of systemic spread of influenza viruses depends on the cleavage of HA0 into HA1 and HA2 subunits and the distribution of appropriate proteases in host tissues. We noticed that the HA0 of H5N1 virus is cleaved very efficiently in human immune cells while the cleavage of the HA0 of H3N2 or H7N9 viruses is inefficient (Figures 3 and 6D). However, externally added TPCK-trypsin, which cleaves the HA0 precursor to HA1 and HA2 subunits, increases the proportion of infected cells in H3N2 and H7N9 infection (Figures 4A,B). There is clear evidence that the multi-basic HA1–HA2 cleavage site constituting of several lysine or/and arginine residues in the HA molecule of the HPAI strains, in contrast to a mono-basic cleavage site in LPAI and seasonal influenza viruses, regulate the pathogenesis of influenza A viruses. The multi-basic cleavage site in the HA has been shown to be a critical determinant of systemic spread of HPAI H5N1 virus (30). The emerged HPAI form of the H7N9 virus expressing the multi-basic site in HA is showing mammalian adaptation with increased pathogenicity and respiratory droplet transmission in ferrets (31). Yet, there must be other factors besides the multi-basic cleavage site that contribute to virus replication and spreading in mammals. Schrauwen and coworkers (32) have shown that the insertion of a multi-basic cleavage site in the HA of H3N2 virus enhances the cleavage of HA and replication in MDCK cells, but it did not increase the pathogenicity in ferrets. In addition, the study by Matthaei et al. (27) shows that the human H5N1 isolates replicate more efficiently in human epithelial cells than the avian H5N1 isolates, suggesting that there must be other differences in addition to multi-basic HA0 cleavage site between human and avian isolates that contribute to the pathogenicity of the virus. Zhao and coworkers have shown that a G158N mutation in HA (H3 numbering) of an avian HPAI H5N1 isolate enhanced viral production and induced stronger host immune responses in mammalian cells (33) which indicates that multi-basic HA1–HA2 cleavage site is not the only factor affecting virulence. Also, our results are consistent with this study of Zhao et al. since both A/Hong Kong/156/1997 and A/Vietnam/1194/2004 H5N1 viruses have asparagine residue at position 158.

It is generally accepted that influenza A virus infection in epithelial cells is productive leading to the release of a great number of progeny viruses from these cells. There is, however, a controversy whether human DCs and Mɸs can efficiently produce infectious influenza A viruses. Bender and coworkers showed that a seasonal H1N1 influenza A virus infection in human DCs is abortive (34). Tate et al. (35) and Ioannidis et al. (36) obtained very similar results in mouse Mɸ and DC model systems with both seasonal H1N1 and H3N2 influenza A viruses. On the other hand, the study by Yu et al. (16) indicates that the HPAI H5N1 virus can replicate productively in human alveolar Mɸs but the infection with seasonal H1N1 virus was abortive. Also, several other studies show that an infection in monocyte-derived Mɸs with seasonal H3N2 or HPAI H5N1 viruses seems to be productive (7, 14, 37, 38). In conclusion, it seems that a H1N1 type influenza A virus infection is likely abortive and, consistent with our results, HPAI H5N1 virus infection is productive (Figures 8A,B) in human immune cells. Nonetheless, there are inconsistent results on the productivity of seasonal H3N2 influenza A virus infection in human cells. In our analyses, a H3N2 influenza A virus as well as the LPAI human isolate of H7N9 virus appeared to cause abortive infection in human moDCs (Figure 8B). Instead, H3N2 and H7N9 virus infections seemed to be productive in Mɸs (Figure 8A). However, the productivity of these viruses was clearly lower when compared with that of the HPAI H5N1 virus. We used another way, namely analyzing the relative amount of virus-specific RNA from cell culture supernatants, to indirectly quantitate the amount of secreted influenza virus into cell culture supernatant. Based on this analysis, there was a good correlation between the amount of infectious virus and the viral RNA levels in the supernatants of H3N2, H5N1, and H7N9-infected Mɸs and moDCs (Figures 8C,D). A technical detail may, however, confound the results since in moDC infections the input virus was not washed away. It may be that, since in the plaque assay of the supernatant samples the background is relative high and H3N2 or H7N9 infections are only weakly productive, we are unable to clearly see an increase in the virus titers. The productivity of influenza virus infections in human DCs and Mɸs have remained poorly investigated and thus our result is a significant addition to the present knowledge.

There is limited amount of information of how many new influenza virus particles or infective units a single-infected cell can produce. Baccam et al. (39) estimated that a single H1N1 virus-infected cell of the upper respiratory tract can produce up to 22 new infectious virus particles leading to a productive infection. It is possible that a single H5N1 virus-infected cell produces even more infective progeny viruses, which may explain the fast and efficient spread of the H5N1 virus in our experimental systems. This study shows that in human immune cells the HPAI H5N1 virus can spread faster than the H3N2 or H7N9 viruses. This may contribute to the ability of H5N1 virus to cause a systemic infection associated with a fatal outcome in H5N1 virus-infected humans.

It is commonly thought that the IFN system is the key factor regulating the efficacy of host antiviral responses and the clearance of the virus. However, the high mortality in H5N1 infection has been associated with an excessive inflammatory response in the lungs of virus-infected individuals (29). Our study shows that the H5N1 virus grows extremely well in human immune cells and it induces strong IFN responses (Figures 1, 2, 9A–C and 10A–C), both of which may contribute to the clinical outcome of the H5N1 infection. Our data show that IFN and also pro-inflammatory cytokine and chemokine responses are higher in infection with H5N1 than with H3N2 or H7N9 viruses at low MOI at the 24 h time point after infection. At the same time, we see that after 24 h infection with the H5N1 virus all cells are infected whereas with the H3N2 or H7N9 virus only proportion of the cells is infected. This suggests that there is a positive correlation between the spread of infection and the cytokine responses. Thus, the strong IFN responses and virus replication are not exclusive of each other; for instance, Ngunjiri et al. (40) have shown that the highly pathogenic H5N1 virus is sensitive to the antiviral actions of IFNs. There is also evidence that H5N1 viruses with high replication competency can ultimately overcome the antiviral effects induced by IFN-α and IFN-β (41). Bordi et al. (42) have shown that IFN-λ and IFN-α have antagonistic antiviral activity against Crimean-Congo hemorrhagic fever virus. However, in our previous study (43) in influenza A virus infection, we did not see any antagonistic activity between IFN-α and IFN-λ, or IFN-λ and IFN-γ, or IFN-α and IFN-γ. We have shown that in H5N1 virus infection IFN-α is produced leading to strong MxA protein expression in the cells which proves that IFN signaling is intact in H5N1-infected cells (12). Still, in this study, we clearly show that HPAI H5N1 virus can replicate and spread extremely efficiently in human innate immune cells despite of strong activation of IFN gene expression and MxA protein expression (Figure 11). This indicates that due to efficient spread of H5N1 virus and high infectivity in the cell cultures there are more cells which can produce IFNs leading to a cytokine storm associated with H5N1-infected patients. This may in part explain the fatal outcome of a H5N1 virus infection in humans. Although the clinical outcome in humans is similar in H5N1 and H7N9 virus infections, surprisingly the antiviral IFN and pro-inflammatory cytokine gene expression is impaired in H7N9 virus-infected immune cells (Figures 9 and 10). Thus, it seems that the mechanism behind the pathogenicity is different in H5N1 and H7N9 virus infection which still demands further investigations.

In this study, we have shown that different types of influenza A viruses may behave very differently in immune cells, primary human monocyte-derived Mɸs and DCs functioning as our model cell systems. While HPAI H5N1 virus efficiently replicated and spread in immune cell cultures seasonal H3N2 and surprisingly LPAI H7N9 viruses showed reduced ability to establish a productive infection. More studies are clearly warranted to further reveal the mechanistic details regulating the ability of HPAI avian influenza viruses to replicate in human primary immune cells and to understand the relationship between the virus and host innate immune system.

## Author Contributions

VW performed most of the experiments, analyzed the data, and wrote the manuscript. SM and PÖ helped with experiments. IJ and PÖ together with VW designed the experiments and wrote the manuscript.

## Conflict of Interest Statement

The authors declare that the research was conducted in the absence of any commercial or financial relationships that could be construed as a potential conflict of interest.
